# A new species of *Mysidopsis* (Crustacea, Mysida) from the Canary and Cape Verde archipelagos

**DOI:** 10.3897/BDJ.12.e139475

**Published:** 2024-12-30

**Authors:** Karl J. Wittmann

**Affiliations:** 1 Zentrum für Public Health, Medizinische Universität Wien, Vienna, Austria Zentrum für Public Health, Medizinische Universität Wien Vienna Austria

**Keywords:** taxonomy, first description, colour variants, key to species, East Atlantic, Macaronesian islands

## Abstract

**Background:**

Within the subfamily Leptomysinae (fam. Mysidae), the tribe Mysidopsini has five here acknowledged genera and 74 extant species. It embraces the genera *Americamysis* with six species from the coasts of the NW-Atlantic (Narragansett Bay to Florida), the Caribbean and Gulf of Mexico; *Brasilomysis* with two species from the SW-Atlantic off Brazilian shores and from the Pacific coast of Ecuador; *Cubanomysis* with three species from the Caribbean, Gulf of California and southern California; *Metamysidopsis* with ten species from the Atlantic coasts of the USA to Brazil, Caribbean, Gulf of Mexico and E-Pacific from California to Panama; and, finally, the globally occurring *Mysidopsis*. Not counting the below-described new species and one fossil species, the latter genus comprises 53 extant species and one non-nominotypical subspecies. With regard to the great number of species, this genus is comparatively homogeneous, not considering the monotypic subgenera *Pseudomysidopsis* and *Mysidopsoides*.

**New information:**

*Mysidopsiscanariensis* sp. nov. is described from five islands of the Canaries and two islands of the Cape Verdes (NE-Atlantic). Records are from depths of 5 to 30 m, mainly over sand, also on stones and rock. It differs from the remaining NE-Atlantic and Mediterranean congeners amongst other features by the lateral margins of the telson distally having a longer bare portion. Amongst these species, it differs from its northern vicariants *M.iluroensis* and *M.gibbosa* in addition by fewer spines on the endopod of uropods. In-situ-photos of *M.canariensis* sp. nov. document at least six strongly different colour variants, four of which are strikingly similar to corresponding variants of *M.jenseni* from the NE-Pacific coast. The latter differs from all species of the E-Atlantic, including the new one, by a mid-dorsal lappet near the caudal margin of the female carapace. A key to the species of *Mysidopsis* from the E-Atlantic and Mediterranean is given.

## Introduction

Wittmann and Wirtz (1998) indicated 16 mysid species and one subspecies sampled in 1994–1995 at the coasts of three Canary Islands, amongst which the authors informally diagnosed five species and the subspecies as potential new taxa. A formal description is presented below for a species reported from the islands of El Hierro and Tenerife by Wittmann and Wirtz (1998) as *Mysidopsis* sp. A (aff.gibbosa). The below-discussed biogeographical importance of this species is further underlined by discoveries in 1996–2003 from three additional islands of the Canary Archipelago and, in 2007, from two islands of the Cape Verde Archipelago. A key to the resulting total of 15 *Mysidopsis* species known from the E-Atlantic and Mediterranean is also provided.

## Materials and methods

### Sampling

Samples were taken during the daytime with diver-operated nets (mesh size 0.5 mm), if not stated otherwise. Boat-operated bottom net (rectangular opening 48.7 cm x 30 cm = 1461 cm^2^, anterior 150 cm with 0.5 mm mesh size, posterior 40 cm with 0.3 mm, 2 kg weights attached) and surface plankton net (same net without weights) were used during the day and night. Geographic position is indicated as decimal coordinates, such as 28.4547N 17.8454W. The material was fixed in 4% formalin and later preserved in an aqueous solution of 80% ethanol with 10% propylene glycol.

### Terminology

Laboratory methods and terminology are outlined by Wittmann (2024b). Terminology of gross structures of the foregut follows Kobusch (1998). Larval stages are termed according to Wittmann (1981). Abbreviations used for the substages of the nauplioid stage are N1 for larvae freshly hatched from the egg membrane, to N4 for those shortly before the moult that leads to the postnauplioid stage. The scheme of diagnosis and the descriptions were adopted from descriptions of *Mysidopsis* species by Wittmann and Griffiths (2018).

### Abbreviations

**BL**, body length measured from tip of rostrum to terminal margin of telson without spines; larvae with rostrum and telson not yet developed were measured simply from anterior to posterior end of the body; **NHMW**, Natural History Museum of Vienna (repository); **OB**, organ of Bellonci; **OP**, ocular papilla = eye papilla; **TFMC**, Museo de Ciencias Naturales de Tenerife (repository); **ZMB**, Zoological Museum of Berlin (repository).

## Data resources

Types and non-types housed in NHMW, TFMC and ZMB.

## Taxon treatments

### Mysidopsis canariensis

Wittmann sp. nov.

CB89B295-4CC8-587F-8985-66030D48F83B

9907E30C-6E2C-4E6B-8FB3-73AAF7EF3CD2

*Mysidopsis* sp. A (aff.gibbosa) – Wittmann and Wirtz (1998): 524 (records from El Hierro and Tenerife); Ulibarri (2013): 5, 52 (in species list of Gran Canaria); Vilas (2015): 10 (in list).

#### Materials

- **Type status:** Holotype. **Occurrence:** recordedBy: Karl J. Wittmann and Peter Wirtz; individualCount: 1; sex: ♂ with BL 4.2 mm; lifeStage: adult; occurrenceStatus: present; preparations: whole animal (in 80% ethanol with 10% propylene glycol); disposition: in collection; otherCatalogNumbers: NHMW-CR-30656; occurrenceID: 0AD83BBB-47BB-57B7-A6FF-CFABA0794CA4; **Location:** islandGroup: Canary Archipelago; island: Gran Canaria; locality: 400 m off the harbor of Pasito Blanco; verbatimDepth: 10–20 m; decimalLatitude: 27.745; decimalLongitude: -15.618; **Event:** samplingProtocol: 19.0°C in 0.5 m depth, on sand, *Cymodocea* and *Caulerpa*; eventDate: 14 Feb 1996; eventTime: 20:20–20:35 LCT, night; habitat: marine sublittoral
- **Type status:** Paratype. **Occurrence:** recordedBy: Karl J. Wittmann and Peter Wirtz; individualCount: 24; sex: 3 ♀♀ ad. with BL 3.3–3.8 mm, 4 ♂♂ ad. 3.7–4.0 mm, 6 ♀♀ subad., 2 ♂♂ subad., 7 imm.; lifeStage: 7 adults, 8 subadults, 7 immatures; occurrenceStatus: present; disposition: in collection; otherCatalogNumbers: NHMW-CR-30657; occurrenceID: 5A25E786-9A61-5A1B-912C-CCEA6665BD29; **Location:** islandGroup: Canary Archipelago; island: Gran Canaria; locality: 400 m off the harbor of Pasito Blanco; verbatimDepth: 10–20 m; decimalLatitude: 27.745; decimalLongitude: -15.618; **Event:** samplingProtocol: 19.0°C in 0.5 m depth, on sand, *Cymodocea* and *Caulerpa*; eventDate: 14 Feb 1996; eventTime: 20:20–20:35 LCT, night; habitat: marine sublittoral
- **Type status:** Paratype. **Occurrence:** recordedBy: Karl J. Wittmann and Peter Wirtz; individualCount: 24; sex: 4 ♀♀ ad. with BL 3.6–4.0 mm, 5 ♂♂ ad. 3.5–4.4 mm, 7 ♀♀ subad., 2 ♂♂ subad., 6 imm.; lifeStage: 9 adults, 9 subadults, 6 immatures; occurrenceStatus: present; disposition: in collection; otherCatalogNumbers: TFMC; occurrenceID: 0E43C9ED-8E24-52DA-8246-298E1F76CFC7; **Location:** islandGroup: Canary Archipelago; island: Gran Canaria; locality: 400 m off the harbor of Pasito Blanco; verbatimDepth: 10–20 m; decimalLatitude: 27.745; decimalLongitude: -15.618; **Event:** samplingProtocol: 19.0°C in 0.5 m depth, on sand, *Cymodocea* and *Caulerpa*; eventDate: 14 Feb 1996; eventTime: 20:20–20:35 LCT, night; habitat: marine sublittoral
- **Type status:** Paratype. **Occurrence:** recordedBy: Karl J. Wittmann and Peter Wirtz; individualCount: 24; sex: 4 ♀♀ ad. with BL 3.6–4.0 mm, 5 ♂♂ ad. 3.5–4.4 mm, 7 ♀♀ subad., 2 ♂♂ subad., 6 imm.; lifeStage: 9 adults, 9 subadults, 6 immatures; occurrenceStatus: present; disposition: in collection; otherCatalogNumbers: ZMB; occurrenceID: F2E3F5BD-5F64-5146-A8BE-466EA35EBB6B; **Location:** islandGroup: Canary Archipelago; island: Gran Canaria; locality: 400 m off the harbor of Pasito Blanco; verbatimDepth: 10–20 m; decimalLatitude: 27.745; decimalLongitude: -15.618; **Event:** samplingProtocol: 19.0°C in 0.5 m depth, on sand, *Cymodocea* and *Caulerpa*; eventDate: 14 Feb 1996; eventTime: 20:20–20:35 LCT, night; habitat: marine sublittoral
- **Type status:** Paratype. **Occurrence:** recordedBy: Karl J. Wittmann and Peter Wirtz; individualCount: 2; sex: 1 ♀ ad. with BL 3.4 mm, 1 imm.; lifeStage: 1 adult, 1 immature; occurrenceStatus: present; disposition: in collection; otherCatalogNumbers: NHMW-CR-30658; occurrenceID: 2C7A4902-1AA6-5450-AB84-6C6DB0EDB9BB; **Location:** islandGroup: Canary Archipelago; island: Gran Canaria; locality: between the Faro de Maspalomas and the harbor of Pasito Blanco; verbatimDepth: 0.5 m; decimalLatitude: 27.732; decimalLongitude: -15.599; **Event:** samplingProtocol: horizontal surface tow, 27.732N 15.599W to 27.745N 15.618W, above bottom depth 15–25 m, 19.0°C in 0.5 m depth; eventDate: 14 Feb 1996; eventTime: 20:20–20:35 LCT, night; habitat: marine surface plankton
- **Type status:** Other material. **Occurrence:** recordedBy: Peter Wirtz; individualCount: 2; sex: 2 ♀♀ ad. with BL 3.8–4.1 mm; lifeStage: 2 adults; occurrenceStatus: present; disposition: in collection; otherCatalogNumbers: TFMC; occurrenceID: 468D624F-1E82-5319-9CC3-D6400A2BB617; **Location:** islandGroup: Canary Archipelago; island: El Hierro; locality: in front of harbor of La Restinga; verbatimDepth: 26 m; decimalLatitude: 27.637; decimalLongitude: -17.984; **Event:** samplingProtocol: stripped with a diver-operated hand net from coarse sand; eventDate: 26 Dec 1994; habitat: marine sublittoral
- **Type status:** Other material. **Occurrence:** recordedBy: Peter Wirtz; individualCount: 1; sex: ♀ with BL 3.7 mm; lifeStage: adult; occurrenceStatus: present; disposition: in collection; otherCatalogNumbers: TFMC; occurrenceID: F9B3A30C-B693-5496-89C3-8BB526D16FD5; **Location:** islandGroup: Canary Archipelago; island: La Palma; locality: Malpique; verbatimDepth: 10 m; decimalLatitude: 28.4547; decimalLongitude: -17.8454; **Event:** samplingProtocol: diver-operated hand net; eventDate: 3 June 2003; habitat: marine sublittoral
- **Type status:** Other material. **Occurrence:** recordedBy: Peter Wirtz; individualCount: 1; sex: ♀ with BL 3.1 mm; lifeStage: immature; occurrenceStatus: present; disposition: in collection; otherCatalogNumbers: TFMC; occurrenceID: 2036A128-EF7D-5F01-943E-0EBC29D62061; **Location:** islandGroup: Canary Archipelago; island: Tenerife; locality: Baja de Adeje; verbatimDepth: 14–18 m; decimalLatitude: 28.099; decimalLongitude: -16.763; **Event:** samplingProtocol: diver-operated hand net, sand; eventDate: 15 Feb 1995; habitat: marine sublittoral
- **Type status:** Other material. **Occurrence:** recordedBy: Karl J. Wittmann; individualCount: 5; sex: 1 ♂ ad. with BL 3.6 mm, 2 imm., 2 juv; lifeStage: 1 adult, 2 immature, 2 juveniles; occurrenceStatus: present; disposition: in collection; otherCatalogNumbers: TFMC; occurrenceID: 64B3F716-57B1-5FE6-BDA7-0491A8BA4296; **Location:** islandGroup: Canary Archipelago; island: Lanzarote; locality: Puerto del Carmen; verbatimDepth: 0.5 m; decimalLatitude: 28.8962; decimalLongitude: -13.7382; **Event:** samplingProtocol: horizontal surface tow along 400 m from 28.8962N 13.7382W to 28.8997N 13.7357W, black sand with algae, 18.9°C in 0.5 m depth; eventDate: 24 May 2001; eventTime: 22:17–22:32 LCT, night; habitat: marine surface plankton
- **Type status:** Other material. **Occurrence:** recordedBy: Karl J. Wittmann; individualCount: 4; sex: 1 ♀ ad. with BL 3.3 mm, 2 ♂♂ ad. 3.8–3.9 mm, 1 juv.; lifeStage: 3 adults, 1 juvenile; occurrenceStatus: present; disposition: in collection; otherCatalogNumbers: ZMB; occurrenceID: D881A234-5A0E-5630-BF02-6E7B9CC3F228; **Location:** islandGroup: Canary Archipelago; island: Lanzarote; locality: Puerto del Carmen; verbatimDepth: 25.0–26.7 m; decimalLatitude: 28.9032; decimalLongitude: -13.7332; **Identification:** dateIdentified: Oct. 2024; **Event:** samplingProtocol: net towed over sandy bottom with algae from 28.9032N 13.7332W to 28.8975N 13.7385W, 18.8°C in 0.5 m depth; eventDate: 24 May 2001; eventTime: 21:56–22:11 LCT, night; habitat: marine sublittoral
- **Type status:** Other material. **Occurrence:** recordedBy: Karl J. Wittmann; individualCount: 5; sex: 3 ♂♂ ad. with BL 3.3–3.6 mm, 1 ♀ ad. 3.7 mm, 1 ♀ subad.; lifeStage: 4 adults, 1 subadult; occurrenceStatus: present; disposition: in collection; otherCatalogNumbers: NHMW-CR-30659; occurrenceID: 6E8A549B-CCAB-51F5-A346-FE1F7FB0FAD5; **Location:** islandGroup: Canary Archipelago; island: Lanzarote; locality: Puerto del Carmen; verbatimDepth: 25.0–26.7 m; decimalLatitude: 28.9123; decimalLongitude: -13.7118; **Event:** samplingProtocol: net towed over black sand and rock with *Cymodocea* and algae from 28.9123N 13.7118W to 28.9148N 13.7048W, 18.7°C in 0.5 m depth; eventDate: 24 May 2001; eventTime: 22:50–23:05 LCT, night; habitat: marine sublittoral
- **Type status:** Other material. **Occurrence:** recordedBy: Karl J. Wittmann; individualCount: 1; lifeStage: immature with BL 3.1 mm; occurrenceStatus: present; disposition: in collection; otherCatalogNumbers: ZMB; occurrenceID: B0B1838D-3BF5-51EE-A481-FCE06C9939DA; **Location:** islandGroup: Canary Archipelago; island: Lanzarote; locality: Puerto del Carmen; verbatimDepth: 23.8–20.4 m; decimalLatitude: 28.9152; decimalLongitude: -13.6323; **Event:** samplingProtocol: net towed over sandy bottom with brown and green algae from 28.9152N 13.6323W to 28.9163N 13.6292W, 19.0°C in 0.5 m depth; eventDate: 24 May 2001; eventTime: 20:32–20:47 LCT, night; habitat: marine sublittoral
- **Type status:** Other material. **Occurrence:** recordedBy: Karl J. Wittmann; individualCount: 1; lifeStage: juvenile with BL 1.3 mm; occurrenceStatus: present; disposition: in collection; otherCatalogNumbers: ZMB; occurrenceID: 8EB22054-2CB4-52E3-B6CD-790344A27ADA; **Location:** islandGroup: Cape Verde Archipelago; island: Santiago; locality: Tarrafal, small bay at plant King Fisher; verbatimDepth: 5–15 m; decimalLatitude: 15.2750; decimalLongitude: -23.7582; **Event:** samplingProtocol: marine rocky shore, material striped from stones with algae, light-supported catch with lamp in front of a diver-operated net, 27°C in 0.5 m depth; eventDate: 18 Aug 2007; eventTime: 19:26–20:38 LCT, night; habitat: marine sublittoral
- **Type status:** Other material. **Occurrence:** recordedBy: Karl J. Wittmann; individualCount: 1; sex: ♀ with BL 2.7 mm; lifeStage: adult; occurrenceStatus: present; preparations: dissected, on 5 slides; disposition: in collection; otherCatalogNumbers: NHMW-CR-30661; occurrenceID: 0183CAB5-CA38-5BB2-971B-920DE5A510C1; **Location:** islandGroup: Cape Verde Archipelago; island: Santiago; locality: Tarrafal, dive point "Arco"; verbatimDepth: 12–22 m; decimalLatitude: 15.2748; decimalLongitude: -23.7602; **Event:** samplingProtocol: marine rocky shore; striped with diver-operated hand net from brown and green algae on rock; brown algae on stones with sand in between; 25°C in 0.5 m depth; eventDate: 19 Aug 2007; eventTime: 16:01–16:56 LCT, day; habitat: marine sublittoral
- **Type status:** Other material. **Occurrence:** recordedBy: Karl J. Wittmann; individualCount: 1; lifeStage: 1 juvenile with BL 1.4 mm; occurrenceStatus: present; disposition: in collection; otherCatalogNumbers: TFMC; occurrenceID: 11A10FAD-E605-52FA-9886-CC64995917F9; **Location:** islandGroup: Cape Verde Archipelago; island: Sal; locality: rocky shore off Palmeira, marine cave at dive point "Palmeira I"; verbatimDepth: 11–21 m; decimalLatitude: 16.7576; decimalLongitude: -22.9889; **Event:** samplingProtocol: taken with a diver-operated hand net inside cave from sandy floor about 20–100 from cave entrance, dimly lit to fully dark, 24°C in 0.5 m depth; eventDate: 26 Aug 2007; eventTime: 10:10–10:40 LCT; habitat: sublittoral marine cave
- **Type status:** Other material. **Occurrence:** recordedBy: Karl J. Wittmann; individualCount: 1; sex: ♀ with BL 3.7 mm; lifeStage: adult; occurrenceStatus: present; disposition: in collection; otherCatalogNumbers: NHMW-CR-30660; occurrenceID: 033B6F01-2F85-5376-9797-8CF49EFCC0FA; **Location:** islandGroup: Cape Verde Archipelago; island: Sal; locality: rocky shore off Palmeira, marine cave at dive point "Palmeira I"; verbatimDepth: 15–22 m; decimalLatitude: 16.7576; decimalLongitude: -22.9889; **Event:** samplingProtocol: taken with a diver-operated hand net inside cave from sandy floor about 20–100 from cave entrance, dimly lit to fully dark, 24°C in 0.5 m depth; eventDate: 26 Aug 2007; eventTime: 12:10–12:55 LCT; habitat: sublittoral marine cave

#### Description

**Figure 1.**
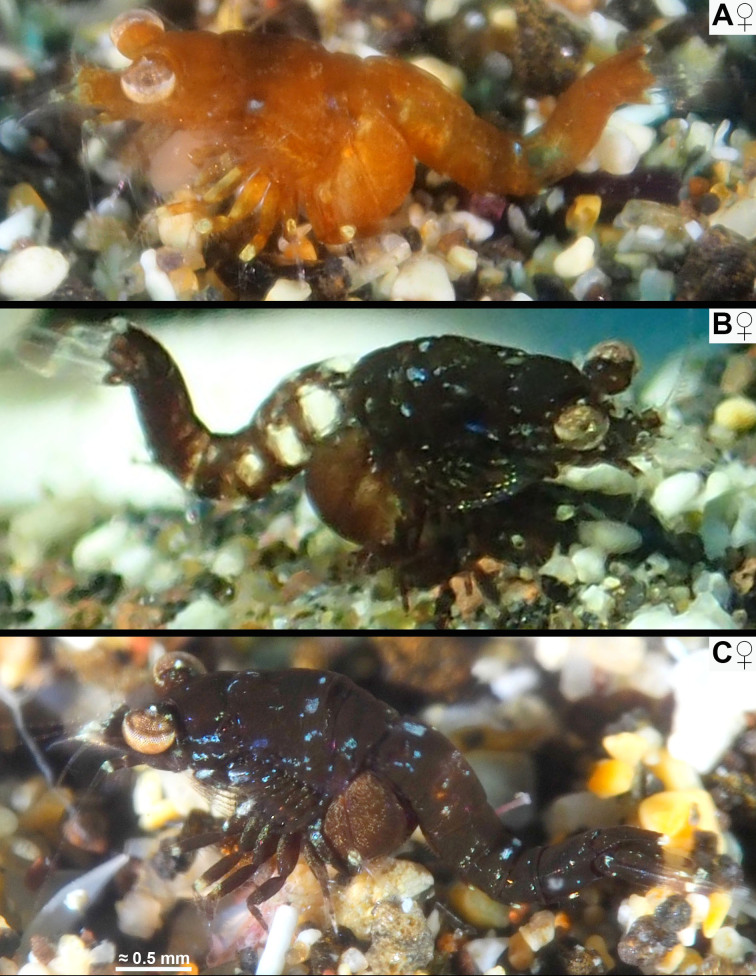
Habitus and colour variants in adult females of *Mysidopsiscanariensis* sp. nov. from shallow sandy bottoms in Lanzarote (**A, C**) and Gran Canaria (**B**). In-situ photos by Dennis Rabeling (A, C) and Sabina López Suárez (B), with permission.

**Figure 2.**
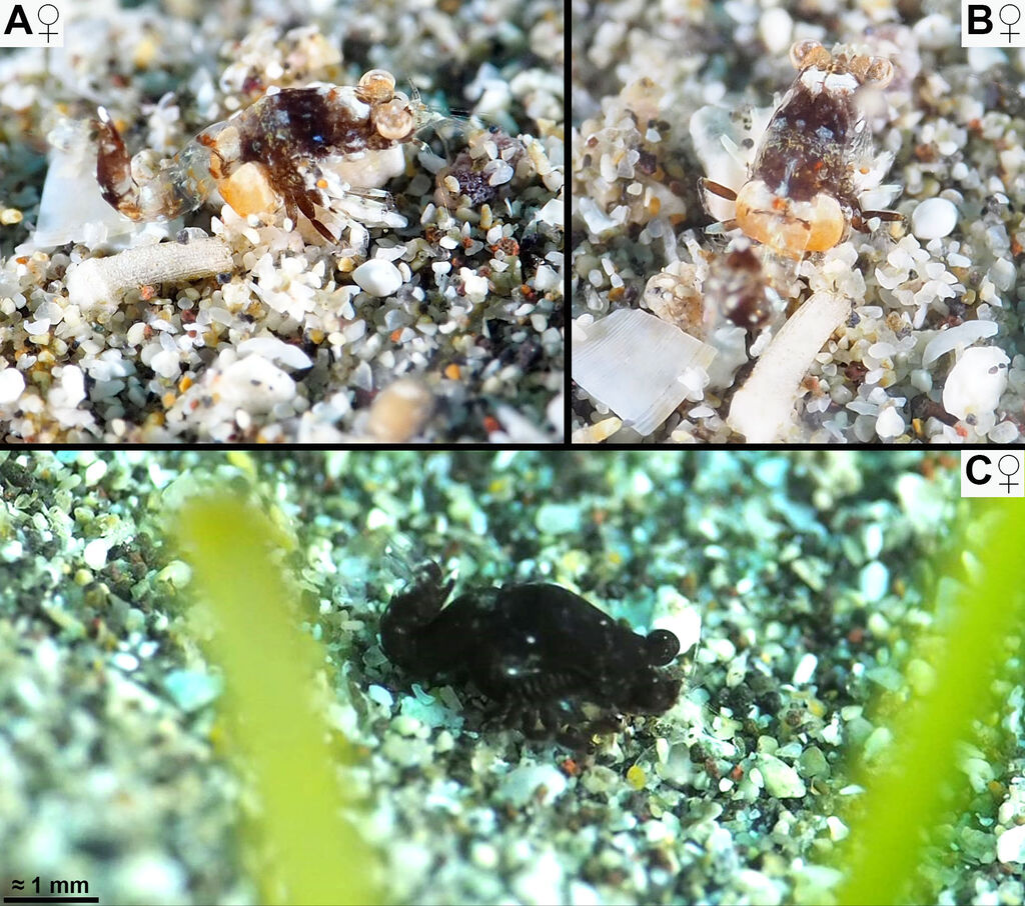
Potentially cryptic (A–B) versus non-cryptic (C) colouring in *Mysidopsiscanariensis* sp. nov. from shallow sandy bottoms in Lanzarote. **A, B** adult female, lateral (A) and dorsal (B) aspects; **C** adult female in lateral view. In-situ photos by Dennis Rabeling, with permission.

**Figure 3.**
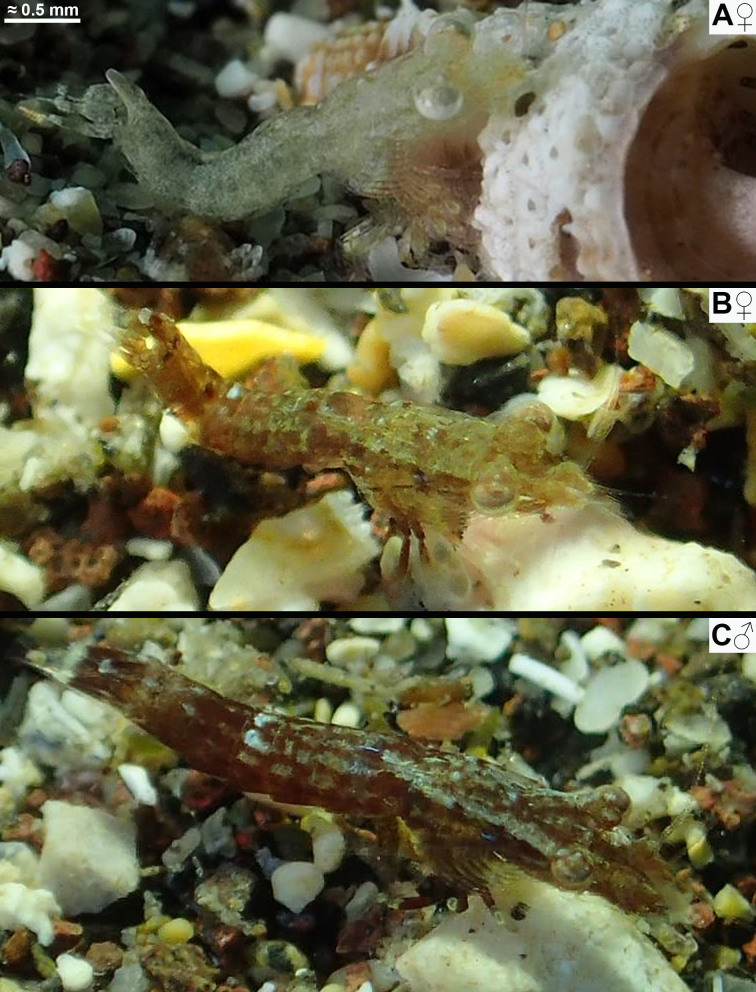
Habitus and colour variants in subadults of *Mysidopsiscanariensis* sp. nov. from shallow sandy bottoms in the Canary Islands. **A–C** lateral aspect of female from Lanzarote (A) and obliquely dorsal aspects of female (B) and male (C) from Gran Canaria. In-situ photos by Dennis Rabeling (A) and Sabina López Suárez (B–C), with permission.

All respective features of the below diagnosis. Body proportions (Figs 1, 2, 3): body length of adults 3.2–4.1 mm (n = 19) in females, 3.3–4.4 mm (n = 21) in the less stout males. Body form stout in females, intermediate in males. Tail upwards bent in immatures to adults, slightly bent between pleomeres 3 and 4 and then more strongly between pleomeres 4 and 5. Flexion increases with body size in both sexes, stronger in females than in same-sized males. Carapace without rostrum 28–35% body length, rostrum 3–6%, cephalothorax 28–35%, pleon without telson 46–54%, telson 11–14% body length. Thoracic sternites normal in both sexes, not reinforced by crossbeams, no sternal processes.

**Figure 4.**
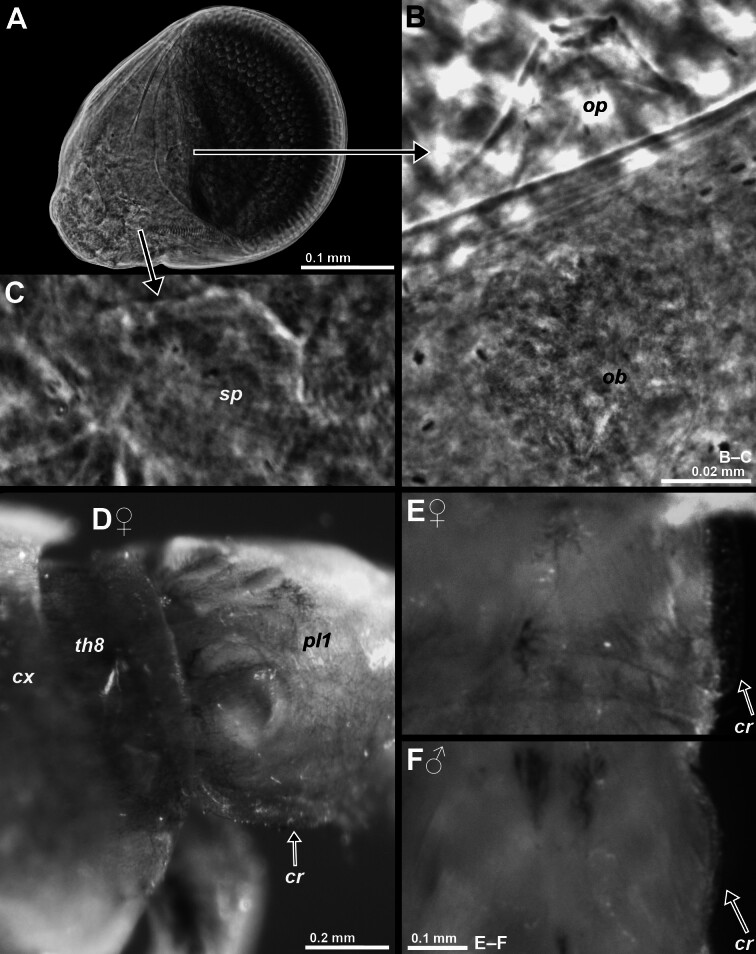
Structure of eyes and first pleomere in adult paratypes of *M.canariensis* sp. nov. from Gran Canaria, males with BL 4.4 mm (A), 3.9 mm (F) and females with 3.9 mm (B–C), 3.6 mm (D–E). **A** right eye, dorsal; **B** ocular papilla and organ of Bellonci of left eye, dorsal; **C** sensory pore organ left eye, dorsal; **D–F** ventro-lateral carina of pleomere 1, lateral (D) and ventral (E–F). A–F, lower-case labels indicate carina (*cr*), carapace (*cx*), organ of Bellonci (*ob*), ocular papilla (*op*), pleomere 1 (*pl1*), sensory pore (*sp*) and thoracomere 8 (*th8*); D–F, pleopods out of frame.

**Figure 5.**
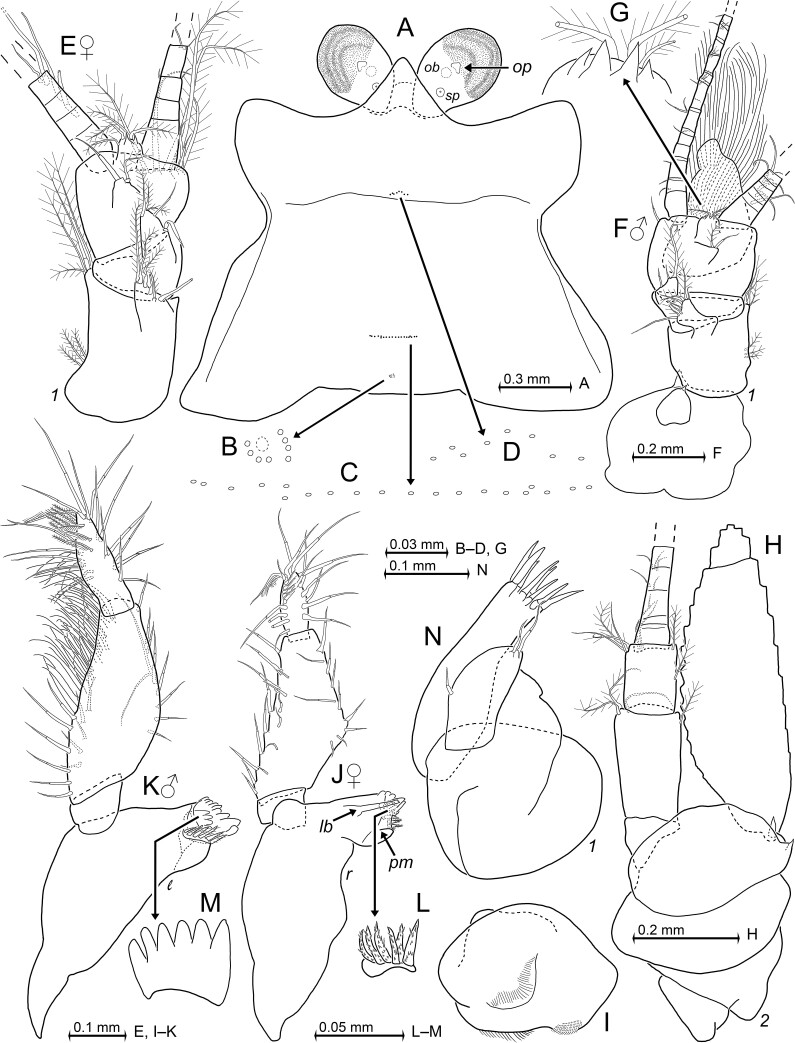
Carapace and cephalic appendages in adult paratypes of *Mysidopsiscanariensis* sp. nov. from Gran Canaria, female with BL 3.9 mm (A–E, H–J, L, N) and male 4.1 mm (F–G, K, M). **A** carapace and eyes expanded on slide, dorsal; **B**–**D** pore groups on carapace; **E** antennula in female, dorsal; **F** antennula and associated lobes from frons in male, dorsal; **G** detail of (F) showing mid-dorsal lobe on terminal segment of antennular trunk; **H** left antenna, proximally with antennal gland and end sac, ventral view, setae of antennal scale omitted; **I** labrum, dorsal = oral face; **J** right mandible in female, caudal, lower-case labels show longitudinal beam (*lb*) and rudimentary pars molaris (*pm*); **K** left mandible in male, rostral; **L, M** details of (J–K) showing right (L) and left (M) digitus mobilis; **N** maxillula, caudal. A–D, pore diameters not to scale; A, J, lower-case labels indicate longitudinal beam (*lb*), organ of Bellonci (*ob*), ocular papilla (*op*), vestigial pars molaris (*pm*) and sensory pore organ (*sp*).

Eyes (Fig. 1, Fig. 4A–C, Fig. 5A): Eyes measure 60–84% antennal scale length or 8–11% body length, distal 50–60% occupied by the cornea. Cornea large, bulbous, calotte-shaped, basal diameter of calotte in dorsal view 23–47% antennal scale length or 7–8% body length. Cornea banded in stacked horizontal planes. The bands represent ellipsoidal to circular, geometric segments, within their plane facing centre of cutting plane of calotte. Eyestalks with smooth surface, dorsally with small sub-conical OP close to mid-proximal margin of the cornea. OP well contrasting as small light conus from darker eyestalk and cornea in living specimens (best visible on left eye in Fig. 1A). Mounting of eyes or comparable techniques are required for firm identification of the small papilla in fixed material. Completely internal organ of Bellonci (OB) associated with external OP (Fig. 4B). Position of OB near papilla is normal in Mysidae species with any papilla; if no papilla present, OB is located mostly near basis of eyestalks (Wittmann 2023, Wittmann 2024a). Diameter of OBs is 41–60 µm at eye length 0.33–0.50 mm (n = 6) in adults of the new species. Individual size data are within range given by Wittmann (2023) for 24 troglophile and 49 trogloxene species of Mysidae. Sensory pore organ (Fig. 4C) located at about half eyestalk length on dorsal face of eyestalk (Fig. 4A and Fig. 5A) in the new species.

Carapace (Fig. 5A–D): Rostrum slightly bent ventrally, extending to 50–80% of length of eyestalks in normal orientation. Disto-lateral edges of carapace broadly rounded (Fig. 5A). Carapace leaving 1 or 2 thoracomeres dorsally exposed. Carapace with 3 mid-dorsal groups of pores: field of 7–12 pores (Fig. 5D) closely in front of cervical sulcus; transversal series of 18–33 pores (Fig. 5C) at 15–20% of carapace length in front of posterior margin; and 8–9 pores surrounding a larger oval structure (Fig. 5B) at 5–6% of carapace length in front of posterior margin.

Antennulae in both sexes (Fig. 5E–G): Short, stout, 3-segmented trunk, stouter in males than in females. Antennulae basally, dorsoventrally (= vertically) flanked by unpaired median processes from frons, a small epi-antennular lobe and a larger hypo-antennular lobe, each with relatively small triangular anterior extension (Fig. 5F). Basal segment of trunk with a dorsal and a lateral setose lobe (apophysis), both lobes not extending beyond median segment. Median segment dorsally with large setose lobe. Dorsal face of terminal segment with mid-terminal lobe (Fig. 5G) with 2–4 acute triangular teeth and 3–5 barbed setae and more proximally somewhat smaller lobe (Fig. 5E and F) with smooth whip-seta and a short, barbed seta.

Antennular trunk of females (Fig. 5E): Basal segment 1.4–1.6 times as long as broad, 47–52% trunk length. Middle segment 18–20%, terminal segment 30–34% trunk length. Flabellum formed by five large plumose setae in ventral position, on mesial half closely behind anterior margin of terminal segment.

Antennular trunk of males (Fig. 5F): Basal segment 1.1–1.3 times as long as broad, contributing 35–40% trunk length, middle segment 19–20%, terminal segment 40–46% trunk length. Terminal segment ventrally with large, strongly setose appendix masculina, longer than combined two distal segments of trunk (basal 2/5 of appendix below drawing plane, there indicated with dashed line in Fig. 5F).

Antennae (Fig. 5H): Sympod with spiniform process on disto-lateral corner and a larger process nearby at dorsal face. Scale without spines, setose almost along all margins, except for smooth margins in most basal portions, outer margin weakly convex, inner margin strongly convex. Apical segment marked by distinct, slightly oblique suture 10–12% of total scale length from tip. Apical segment with five large, plumose setae. Antennal flagellum laterally (Fig. 1B) and backward curved in loco (unlike in mounted antenna in Fig. 5H). Scale extends 1/3 of its length beyond 3-segmented peduncle. Basal segment 14–23% peduncle length, second 45–51%, third 27–35% peduncle length.

Labrum (Fig. 5I): Labrum well-rounded, aborally = dorsally with short anteriorly projecting, rounded bulge. Masticatory surface poorly cuticularised; oral face with mid-ventral field of fine hairs; caudally with paramedian fields of stiff bristles facing mouth field.

Mandibles (Fig. 5J–M): Masticatory parts strongly differ between left and right mandibles, but do not show obvious sexual dimorphism. Processus incisivus of left mandible (Fig. 5K) with three strong plus 4–6 small teeth, no longitudinal beam; lacinia mobilis strong, with six thick, distally rounded, smooth teeth; pars centralis with 5–6 densely set, serrated spine-like teeth, decreasing in size proximally, pars centralis with proximal brush of stiff bristles; no processus molaris. Processus incisivus of right mandible (Fig. 5J) supported by a longitudinal beam, this processus with total of two strong plus five small teeth; lacinia mobilis (Fig. 5L) with six comparatively slender, serrated teeth; pars centralis with 3–4 subequal, weakly serrated spine-like teeth plus bristles; processus molaris unarmed, reduced to a small lobe.

Palp 3-segmented with segments 1–3 counted from basis contributing 12–17%, 57–63%, 24–28% to total palp length in females, 11–18%, 52–59%, 30–34% in males. Palp with small basal segment smooth along all margins; both remaining segments with more setae which are more densely set in males than in females. Males with >30 smooth setae densely set on distal half of the lateral margin of the median segment (Fig. 5K), females with <10 setae loosely set along corresponding stretch (Fig. 5J). Terminal segment mostly with smooth setae, but distal half with moderately long, modified seta (central portions bilaterally with acute barbs). This seta in males proximally accompanied by 6–8 shorter setae with shorter acute barbs bilaterally on distal 80% seta length; in females accompanied by 5–6 short setae with short barbs bilaterally on distal 60% seta length. No soft barbs on these setae in both sexes.

**Figure 6.**
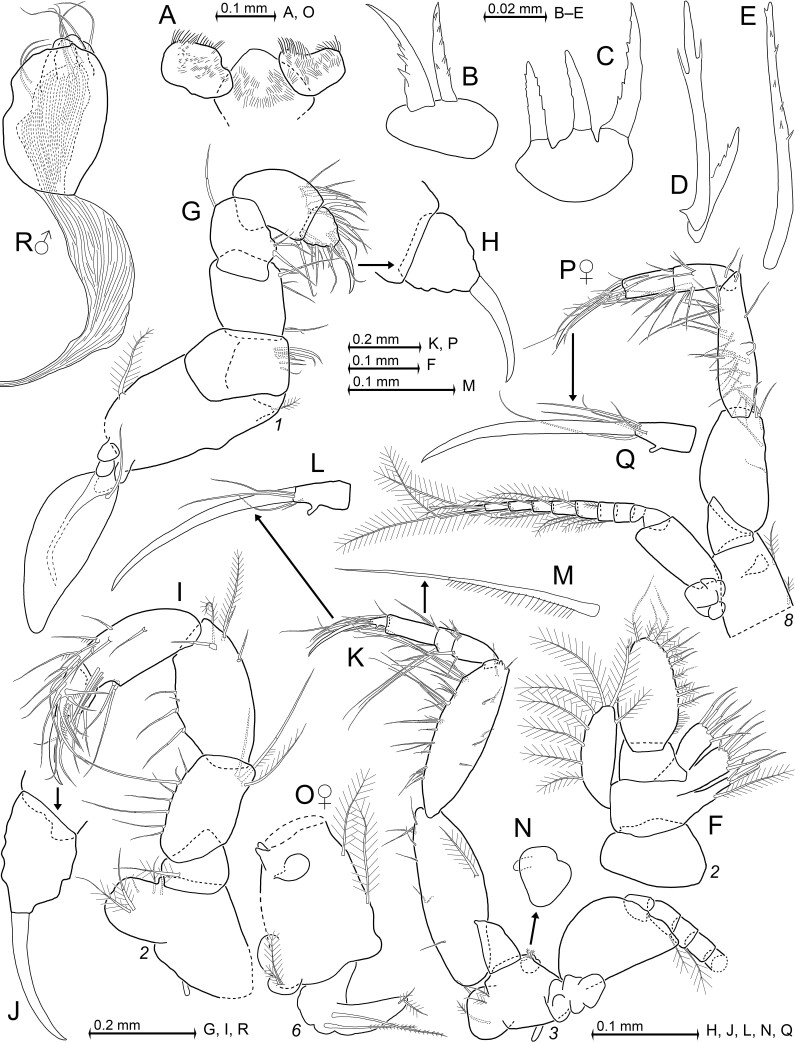
Thoracopods, penes and some cephalic features in adult paratypes of *Mysidopsiscanariensis* sp. nov. from Gran Canaria, male with BL 4.1 mm (A–N, R) and female 3.9 mm (O–Q). **A** labium with part of sternite, ventral; **B**–**E** modified spines of foregut, namely from dorsolateral infoldings (B) and from anterior (D–E) and posterior (C) parts of lateralia; **F** maxilla, caudal, broken parts of setae here supplemented from bilaterally opposite maxilla and indicated with dashed lines; **G** thoracic endopod 1 with epipod, caudal, exopod omitted; **H** detail of (G) showing dactylus with claw, setae omitted; **I** thoracic endopod 2, rostral; **J** detail of (I) showing dactylus with nail, setae omitted; **K** thoracopod 3, caudal; **L**–**N** details of (K) showing dactylus with claw (L), modified seta from median segment of carpopropodus (M) and lobe from disto-lateral edge of basis, in this case visible through bleached sympod (N); **O** thoracic sympod 6 with rudimentary oostegite, rostral face; **P** thoracopod 8 in ♀, caudal, oostegite omitted; **Q** detail of (P) showing dactylus with nail; **R** penis, caudal.

Labium (Fig. 6A): Paragnaths and corresponding part of sternite with fields of fine hairs. Mesial face (upper margin in Fig. 6A) of paragnaths with stiff bristles.

Foregut (Fig. 6B–E): Gross structure as listed by Kobusch (1998): table 1 for *Mysidopsisgibbosa* G.O. Sars 1864. Besides many setae and simple spines, there are modified spines on lateralia and on dorso-lateral infolding in the present species: anterior part of lateralia with many apically pronged spines in diverse modifications (Fig. 6D and E), in part bearing small denticles along distal half and a few short unilaterally serrated spines (Fig. 6D); posterior part of lateralia on each side with cluster of three weakly-serrated spines (Fig. 6C); dorso-lateral infolding on each side with closely-set pair of spines unilaterally armed with strong toothlets (Fig. 6B).

Maxillulae (Fig. 5N): Terminal segment moderately slender, transversely truncate apex with 8–9 moderately strong, smooth spines, no setae. Endite large, apically with one or two smooth whip setae; one additional seta of that type on lateral face, roughly half-way from basis.

Maxillae (Fig. 6F): Second segment of maxillary sympod with three setose endites, each with numerous smooth, some of which spine-like, whip setae on distal margin. Only most proximal endite with additional, large, plumose seta. Exopod slender, reaching to 0.2–0.4 length of terminal segment of endopod. Exopod with total of 8–10 plumose setae mostly along lateral margin; apex with large seta, not in continuous series with setae on lateral margin; no setae on proximal 80–90% of mesial margin. Palp comparatively large, 2-segmented; terminally evenly rounded. Distal segment 1.8–1.9 times longer than maximum width, contributing 70–80% to total palp length; terminus about evenly rounded. This segment with intermediate-sized whip setae on terminal margin and with shorter whip setae on distal 3/5 of mesial margin; all setae with bilaterally barbed handle and smooth flagellum; proximal portion of mesial margin with tiny hairs. No setae on short proximal segment or on lateral margin of distal segment.

Thoracopods 1–8 (Fig. 1C, Fig. 6G–Q): Coxal (= most basal) portion of sympods 2–3 with small linguiform lobe (Fig. 6I and K). First thoracic epipod large, linguiform, distally rounded, sub-basally with single smooth seta. Size measured from praeischium to dactylus increases from endopod 1 to 6 and then decreases from 6 to 8 in both sexes (Fig. 1C, Fig. 6I, K and P). Claws 1–2 strong, curved, with thick basis Fig. 6H and J), claws 3–8 more slender, less curved, needle-like (Fig. 6L and Q). Rectilinear measurement of curved claws increases from endopods 1 to 5 and decreases from 5 to 8. Sizes of basal plates and flagellae increase from exopod 1 to 4–6 and then decrease from 6 to 8 in both sexes. Basal plates with distal and lateral margins converging at about 80 angular degrees, disto-lateral edge rounded. Flagellae of exopods 1, 8 with eight segments, flagellae 2–7 with nine segments, not counting intersegmental joint between basis and flagellum.

Thoracic endopod 1 (Fig. 6G and H): Coxa with large barbed (almost plumose) seta, positioned latero-ventrally at some distance from insertion of exopod (omitted in Fig. 6G). Coxa mesially with small conical lobe with small barbed seta at apex. Basis mesially with 5–6 smooth whip setae, laterally without setae. Ischium and merus fused as single element. Merischium only 1.1 times carpus length, this being much shorter than combined propodus plus dactylus. Tarsus normal, comparatively stout, with separate carpus, propodus, dactylus and claw, segments not subdivided. Tarsus with smooth setae only, namely mostly whip setae; dactylus densely setose.

Thoracic endopod 2 (Fig. 6I and J): Segmentation interpreted in analogy to endopods 3–8. Basis with large endite bearing two medium-sized and three short whip setae, each with bilaterally barbed handle. Praeischium with 1–3 medium-sized to large seta of that type. Ischium and merus separate. Ischium 0.7–0.8 times merus length, merus 1.1–1.2 times carpopropodus. Ischium with 6–8 smooth whip setae on mesial margin and 2–3 barbed setae near lateral margin on distal half. Merus with 3–4 smooth whip setae on mesial margin, three such setae plus two plumose setae somewhat proximally from disto-lateral edge. Carpopropodus with angular constriction to 75–85% width, reaching from 78–89% carpopropodus length to articulation with dactylus (Fig. 6I). Distal 2/3 of carpopropodus with many smooth whip setae; disto-lateral 1/5 with additional 2–3 whip setae with barbed handle. Dactylus longer than wide (Fig. 6J), strongly setose including longitudinal row of setae, each being 'serrated' by double series of stiff barbs along proximal 1/2 to 2/3. Width and, accordingly, also stiffness of barbs increases with distal position of the setae.

Thoracic endopods 3–8 (Fig. 6K–Q): Basis (fused with sympod) of endopods with crescent-shaped to subcircular, soft lobe (lappet) on rostral face near disto-lateral edge (Fig. 6K, O and P). This lobe with short teat-like process in endopod 3 (Fig. 6N). Basis 3 with distinct endite bearing 2–3 barbed setae and one smooth whip seta; this endite smaller than that of basis 2. Indistinct endite in bases 4–5, no endite in bases 6–8. Pra­eischium 3–8 normal, without setae. Ischium and merus separate. Length of ischium and tarsus decrease, whereas length of merus increases from endopod 3 to 8. Ratio of merus length to ischium length increases weakly from 0.8–0.9 in endopod 3 to 1.1–1.2 in endopod 8. Ischium 3 with several smooth whip setae along mesial margin, one large plumose seta on caudal face close to middle of lateral margin. Merus 3 along mesial margin with six clusters of 4–8 smooth whip setae each; 3–4 stand-alone setae of that type on caudal face, mostly at some distance from each other along lateral margin, additional three such setae in analogous position on rostral face. Meri 3–7 with 1.2–1.5 times carpopropodus length, merus 8 with 1.3–1.5. Carpopropodi 3–7 with three segments (Fig. 6K); middle segment 0.3–0.6 times length of other segments. Carpopropodus 3 besides many smooth whip setae with unilaterally barbed setae, two short ones on basal segment and a long one (Fig. 6M) on short median segment. Only smooth setae on carpopropodi 4–8 and on meri plus dactyli 3–8. Carpopropodi 3–8 terminally with 1–2 pairs of large, smooth, paradactylary setae. Dactyli 3–8 with minute evagination on inner margin, with weakly-curved needle-like claw and 4–5 normal smooth setae (Fig. 6L and Q).

Penes (Fig. 6R): Stout, only 1.3–1.5 times longer than maximum width; length 50–60% merus length of thoracic endopod 8.

**Figure 7.**
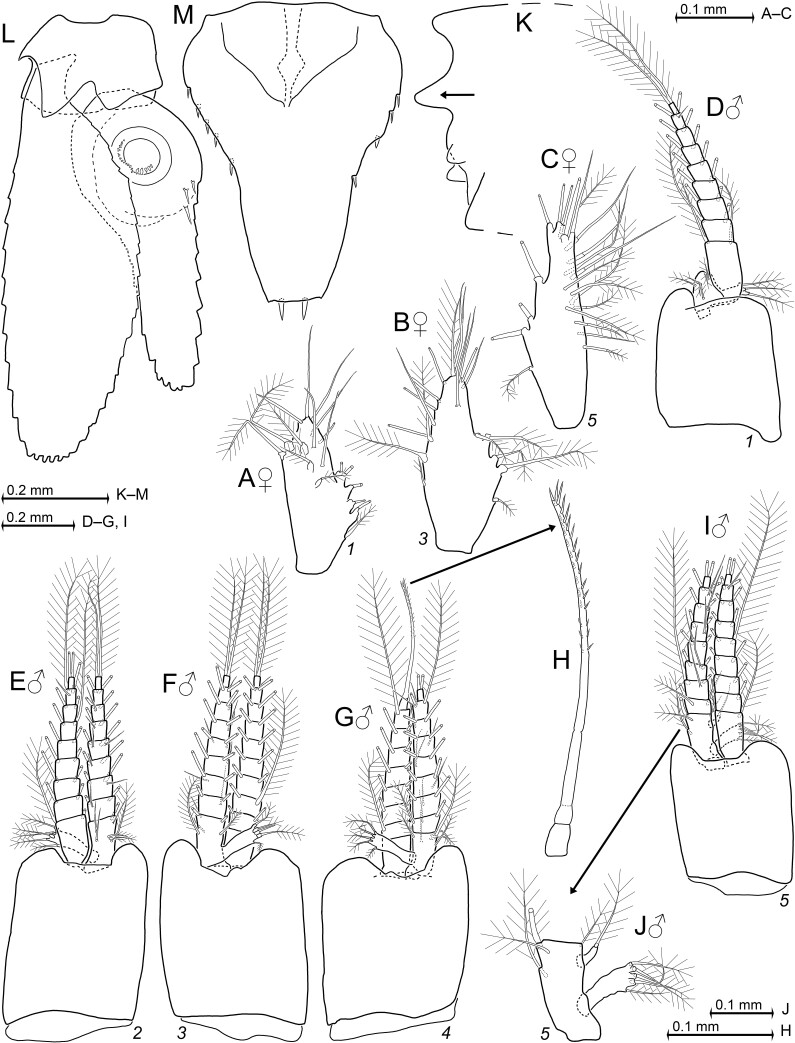
Pleopods and tail fan in adult paratypes of *Mysidopsiscanariensis* sp. nov. from Gran Canaria, female with BL 3.9 mm (A–C, K) and male 4.1 mm (D–J, L–M). **A**–**C** right pleopods 1, 3, 5 in female, rostral = lateral (A–B) and caudal = mesial (C); **D**–**G** pleopods 1–4 in male, rostral (D–E) and caudal (F–G); **H** detail of (G) showing modified seta on terminal segment of exopod 4; **I** pleopod 5 in male, rostral; **J** detail of (I) showing basal segment of endopod 5, rostral; **K** caudal margin of pleomere 6, lateral, arrow points to scutellum paracaudale; **L** uropods, ventral; **M** telson with anal lobe, ventral.

Pleon (Figs 1, 3): Pleomeres 1–6 contribute 16–21%, 15–18%, 14–16%, 12–14%, 11–14% and 20–31%, respectively, of total pleon length. Pleomeres 1–5 are 0.5–1.0, 0.5–0.9, 0.5–0.8, 0.4–0.7 and 0.4–0.7 times length of sixth pleomere. Each pleomere bilaterally with longitudinal ventro-lateral carina decreasing in width caudally. Carinae running essentially horizontally from anterior to posterior margin of pleurites, with weak ventral inclination in sagittal plane (Fig. 4D). Only carina 1 of adult females with about twice the width (Fig. 4E) of carina in adult males (Fig. 4F) and in non-adults of both sexes. Scutellum paracaudale (arrow in Fig. 7K) subtriangular with well-rounded tip, dorsal margin concave, ventral margin convex.

Pleopods (Fig. 7A–J): Female pleopods (Fig. 7A–C) with indistinct or absent endopodal lobe; pleopod size increasing caudally. Male pleopods (Fig. 7D–J): all sympods subrectangular, no setae. Sympods 2–4 (Fig. 7E–G) large, subequal; sympod 1 (Fig. 7D) about same-sized as sympod 5 (Fig. 7F), each smaller than sympods 2–4. Exopod 1 is 4 or 5 times length of endopod, whereas exopods 2–5 are about as long as the corresponding endopods. Subrectangular, laterally directed, pseudobranchial lobe from basal segment of endopods 1–5, each distally with 5 small, barbed setae. Small accessory lobe from endopod 5 with one barbed seta at apex (Fig. 7J). Each segment of endopods 2–5 with two large, plumose setae on or close to distal margin; basal segment with additional 1–4 smaller, barbed or plumose setae on mesial margin. Basal to penultimate segments of endopods 2–4 each with one or two small, smooth setae on rostral face; one such seta on second to penultimate segment in endopod 5; all endopods without such setae on terminal segment. Almost every segment of exopods 1–5 with two large, plumose setae on distal margin, except terminal segment of exopod 4, which bears a large modified seta on tip, but no additional seta (Fig. 7G–H). All except exopod 4 show 1–(2) small smooth seta in subterminal position on rostral face of penultimate segment.

Tail fan (Fig. 7L and M): Sympod of uropods 0.3–0.4 times telson length, endopod 0.9–1.1, exopod 1.2–1.4; exopod 1.2–1.4 times endopod (Fig. 7L). Exopod with weakly-convex outer and more strongly convex inner margins, setose along all margins; comparatively stout, length 2.5–3.5 times maximum width. Statolith composed of fluorite, diameter 87–111 µm; habitus discoidal, with prominent tegmen, concave fundus; statolith formula 2 + 2 + 1 + (0–2) + (4–7) + (3–6) = 14–19 (n = 6). Telson length 1.1–1.7 times maximum width (Fig. 7M), 0.8–1.0 times length of last pleomere. Spaces between lateral spines increase caudally, partly in discontinuous series. Additional details of uropods and telson in diagnosis.

**Figure 8.**
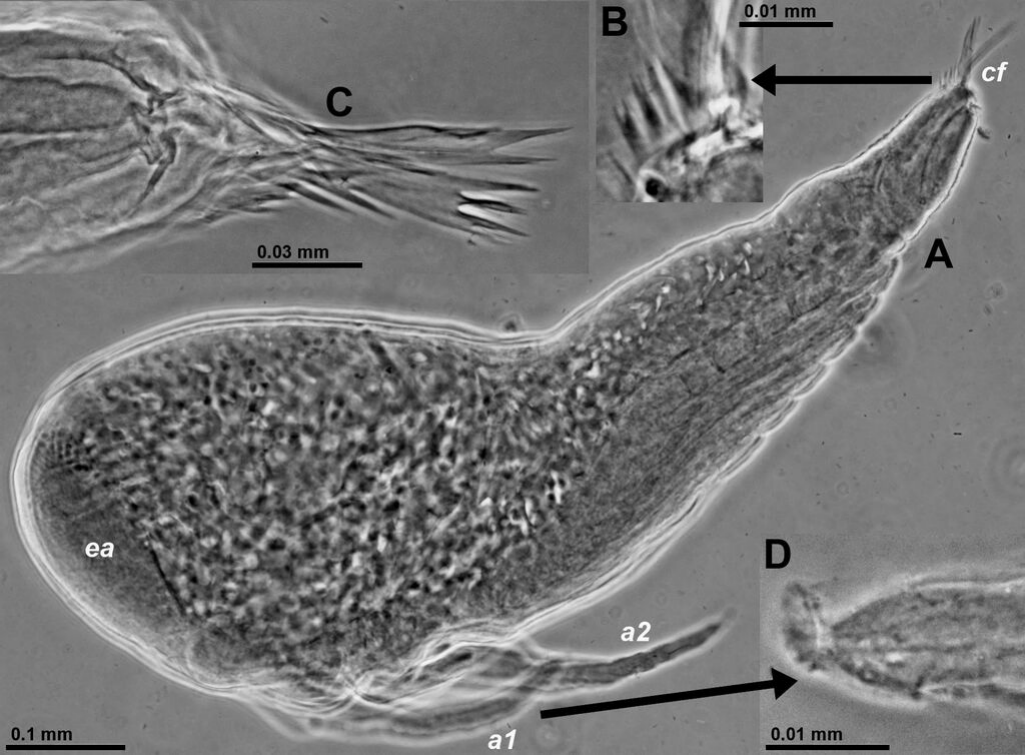
Nauplioid larvae at substage N3 in *Mysidopsiscanariensis* sp. nov. from Lanzarote. **A** habitus, lateral, lower-case labels indicate antennula (*a1*), antenna (*a2*), caudal furca (*cf*) and eye anlage (*ea*); **B** detail of (A) showing series of spines flanking caudal furca (the latter out of focus); **C** tip of abdomen with caudal furca flanked by series of spines in another specimen, lateral; **D** tip of antennula in yet another specimen. All specimens taken from marsupium of female with BL 3.3 mm.

Nauplioid larvae (Fig. 8): Female with BL 3.3 mm carrying five nauplioid larvae at substage N3 with BL 0.84–0.96 mm. Abdomen ends in a caudal furca (Fig. 8A and C) formed by pair of small lobes each bearing one large furcal spine, distal third of the latter multi-furcate (Fig. 8C). Caudal furca is dorsally and ventrally flanked by series of spines decreasing in size proximally, with largest spines bifurcate (Fig. 8B). Such series has, so far, not been described in any species of the tribus Mysidopsini. Both series are proximally followed by several small subequal spines (out of focus in Fig. 8A and C). Tip of antennula and antenna furnished with 5–8 minute triangular scales (teeth ?; Fig. 8D).

#### Diagnosis

Rostrum well developed, triangular, apically forming a 40–90° angle, tip rounded; slightly bent ventrally, extending to basal segment of antennular trunk. Carapace with well-marked cervical sulcus; without mid-dorsal protuberance; caudal margin overall concave with straight to slightly convex median portion. Eyes large, eyestalks well developed, with large bulbous functional cornea; ocular papilla < 1/5 cornea diameter (identification of the papilla may require mounting on slides). Antennal scale length 2.6–3.7 times maximum width. Scale extends 0.2–0.4 times its length beyond antennular trunk; scale with distinct apical segment. Left mandible without processus molaris, right mandible with small, reduced processus; palp normal, 3-segmented, larger and more setose in males than in females. Sympods of maxillula and maxilla without setose expansion. Carpopropodi of thoracic endopods 3–7 each with three segments, middle segment always much shorter than the other two segments, carpopropodus 8 with two or three segments. Endopods 1–2 terminally strongly setose, strong claw throughout; endopods 3–8 apically less strongly setose, with thin needle-like claw. Marsupium normal, thoracopod 6 with small, but distinct, linguiform oostegite. Penes well developed, tubular, stout, each penis with three lobes and several smooth setae flanking ejaculatory opening. Female pleopods reduced, rod-like, setose. All male pleopods biramous, densely setose, with large subrectangular sympod. Endopods 1–5 with 1, 9, 9, 9 and 9 segments, respectively; basal segment caudally with large, subrectangular exite (pseudobranchial lobe); basal segment of only endopod 5 with small additional exite. Exopods 1–5 with 9, 9, 10, 9 and 9 segments. Terminal segment of exopod 4 with large modified seta. Distal half of this seta with bilateral series of short, acute barbs. Endopod of uropods with 3–4 spines on section between 30% and 50% distance from basis, close to mesial margin below statocyst in both sexes; spines increasing in length distally. Endopod extending 40–59% of telson length beyond telson, exopod 68–91%. Telson trapeziform with sigmoid lateral margins and transversal terminal margin; disto-lateral edges angular, distally slightly produced. Each lateral margin with 3–5 small spines along the middle section reaching from 20–25% to 50–60% distance from basis, remaining sections bare. Terminal margin with paramedian pair of larger spines with 6–8% telson length, no additional spines. Telson with total of 8–12 spines in both sexes.

#### Etymology

The species name is a Late Latin adjective with feminine ending, once used as an attribute for inhabitants of the Canary Islands.

#### Distribution

Type locality is Gran Canaria, 400 m off the harbour of Pasito Blanco, 27.745N 15.618W, 10–20 m depth. The species so far known from the Canaries (islands El Hierro, La Palma, Tenerife, Gran Canaria and Lanzarote) and the Cape Verdes (islands Santiago and Sal), 15–29° N, 14–24° W, being epibenthic during the daytime in 5–30 m depth (photos in Figs 1, 2, 3 from 5–10 m), mostly on sandy bottom, also on stones and rock; these substrates in part with *Cymodocea* and algae. Excursions by Peter Wirtz and the present author to Madeira, to the coast of Senegal (West Africa) and to the Gulf of Guinea did not yield this species.

## Identification Keys

### Key to the species of *Mysidopsis* from the East Atlantic and Mediterranean

**Table:** 

1	Telson with unarmed V-shaped cleft penetrating 1/10 telson length. NE-Atlantic: south-western Norway to coast of Portugal, Baltic, entire Mediterranean, Marmora Sea, 2–400 m	***M.angusta* G.O. Sars, 1864**
–	Telson entire or with minute rounded median emargination	2
2	Carapace with one or two mid-dorsal protuberance(s)	3
–	Carapace without mid-dorsal protuberance (Fig. 3C)	6
3	Carapace with only one mid-dorsal protuberance; rostrum produced with angle slightly narrower than rectangular; telson linguiform, distally continuously rounded	5
–	Carapace with two mid-dorsal protuberances; rostrum short, wide-angled triangular; telson trapezoid with rounded disto-lateral edges	4
4	Carapace with two closely adjoining mid-dorsal protuberances behind cervical sulcus, followed by longitudinal saddle; median segment of mandibular palp with large triangular, lateral expansion armed with two spines (only female known). Atlantic coast of South Africa: False Bay, 10–29 m	***M.camelina* O.S. Tattersall, 1955**
–	Carapace with large mid-dorsal hump closely behind cervical sulcus and with smaller hump shortly in front of posterior margin, no saddle behind; median segment of mandibular palp without lateral expansion. NE-Atlantic from North Sea to coast of Morocco, entire Mediterranean and Marmora Sea, 0.2–179 m	***M.gibbosa* (G.O. Sars, 1864)**
5	Telson extending beyond endopod of uropods; endopod with 9 subequal slender spines; antennal scale undivided (only female known). Atlantic coast of South Africa: Lambert’s Bay, coastal	***M.eremita* O.S. Tattersall, 1962**
–	Telson falling far short of endopod of uropods; endopod with 10–18 spines increasing in length caudally; antennal scale with short, but distinct apical segment. Atlantic coast of South Africa: False Bay, Cape Peninsula, 4–10 m	***M.zsilaveczi* Wittmann and Griffiths, 2014**
6	Lateral margins of telson with spines	8
–	Telson with bare lateral margins	7
7	Telson subtriangular, its narrow terminal margin with very small median emargination flanked by only one pair of long spines; uropods without spines. Atlantic and Indian Ocean coasts of South Africa: Table Bay to Algoa Bay, 5–7 m	***M.bispinosa* O.S. Tattersall, 1969**
–	Telson trapezoid, with three pairs of spines at transverse, slightly convex, terminal margin; paramedian pair longest, flanking very small emargination, neighbouring pair one-third shorter, disto-lateral pair minute; endopod of uropods with two unequal spines. Atlantic coast of South Africa: Langebaan Lagoon, False Bay, 7–9 m Telson trapezoid, with three pairs of spines at transverse, slightly convex, terminal margin; paramedian pair longest, flanking very small emargination, neighbouring pair one-third shorter, disto-lateral pair minute; endopod of uropods with two unequal spines. Atlantic coast of South Africa: Langebaan Lagoon, False Bay, 7–9 m	***M.suedafrikana* O.S. Tattersall, 1969**
8	Telson linguiform to trapezoid	11
–	Telson subtriangular	9
9	Endopod of uropods with > 2 spines near statocyst region	10
–	Endopod of uropods with single long spine below statocyst near mesial margin. N-Atlantic: Iceland, Faroe Islands; E-Atlantic: Norway to Portugal; entire Mediterranean, 13‒700 m	***M.didelphys* (Norman, 1863)**
10	Rostrum long, triangular, with rounded tip; endopod of uropods ventrally with 19–31 spines along sub-basal to subterminal portions of mesial margin. Atlantic and Indian Ocean coasts of southern Africa: Namibia, Radford Bay; South Africa: Langebaan Lagoon to Algoa Bay, 4–35 m	***M.similis* (Zimmer, 1912)**
–	Rostrum short, truncate with rounded or less often broadly sinusoidal latero-terminal edges. Endopod of uropods with 5–7 spines in series with distally increasing size on proximal half of mesial margin near statocyst. Atlantic coast of South Africa: False Bay, Cape Peninsula, 4–6 m	***M.abbreviata* Wittmann and Griffiths, 2018**
11	Endopod of uropods ventrally with spines along mesial margin between statocyst and 15–50% endopod length from tip	13
–	Endopod of uropods ventrally with spines along mesial margin between statocyst and 0–6% endopod length from tip	12
12	Lateral margins of telson all along with dense series of about 40 short spines; endopod of uropods with about 30–50 spines in dense series between statocyst and tip. Atlantic and Indian Ocean coasts of southern Africa: Namibia, Radford Bay; South Africa: Lambert’s Bay, False Bay, Algoa Bay, 5–35 m	***M.schultzei* (Zimmer, 1912)**
–	Telson with 19–20 spines only on distal half of each lateral margin, proximal half bare; endopod of uropods with 34–36 short spines in dense series between statocyst and 6% endopod length from tip. NE-Atlantic: Bay of Biscay: Le Danois Bank, 828 m	***M.cachuchoensis* San Vicente, Frutos and Sorbe, 2013**
13	Each lateral margin of telson with about 50 small spines; endopod of uropods with about 40–50 spines; rostrum large, triangular with pointed apex, reaching to distal margin of basal segment of antennular trunk; telson terminally broadly rounded. Atlantic and Indian Ocean coasts of southern Africa: Namibia, Lüderitz Bay; South Africa: Great Berg Estuary, False Bay, Algoa Bay, 0–12 m	***M.major* (Zimmer, 1912)**
–	Each lateral margin of telson with < 12 spines; endopod of uropods with < 10 spines	14
14	Each lateral margin of telson with 8–10 short spines; endopod of uropods with 6–8 spines; rostrum horizontally produced, triangular with pointed apex reaching half-length of basal segment of antennular trunk; telson linguiform, terminally truncate with broadly rounded disto-lateral edges. Mediterranean: Balearic Sea: off Mataró, 17–21 m	***M.iluroensis* San Vicente, 2013**
–	Each lateral margin of telson with 3–5 small spines; endopod of uropods with 3–4 spines; rostrum triangular, apically forming 40–90° angle, tip rounded, reaching basal segment of antennular trunk; telson trapeziform with angular disto-lateral edges. E-Atlantic: Canary Islands (El Hierro, La Palma, Tenerife, Gran Canaria, Lanzarote) and Cape Verde Islands (Santiago, Sal), 5–30 m	***M.canariensis* Wittmann sp. nov.**

Keys of San Vicente (2013) and Wittmann and Griffiths (2018), merged, modified and updated.

## Discussion

### Validity of the new species

*Mysidopsiscanariensis* sp. nov. differs from its northern vicariant *M.gibbosa* G.O. Sars, 1864, by the flat carapace without humps (Fig. 5A; vs. two humps), by eyestalks with shorter OP (Fig. 4B and Fig. 5A; length < 1/5 cornea diameter vs. 1/5 to 1/4) and by lateral margins of the telson distally with longer bare portion (Fig. 7M; 1/3 to 1/2 vs. 1/6–1/4). It shares the flat carapace, eyes with minute papilla and terminally transversely truncate, trapezoid telson with only three species amongst the total of 53 extant species (not including the new one) currently acknowledged in this genus: *M.juniae* da Silva, 1979, *M.suedafrikana* O.S. Tattersall, 1969 and nearly also *M.iluroensis* San Vicente, 2013, as discussed in the following:

*Mysidopsisjuniae* from shallow waters at the southeast coast of Brazil, 23–26°S (Vaz 2012, Miyashita and Calliari 2016), differs from the new species by a telson with bare lateral margins (vs. with 3–5 spines each), terminal margin transverse with vs. without mid-terminal protrusion between a pair of disto-sublateral spines; endopod of uropod with about 14–18 vs. 3–4 spines between statocyst and 1/3 vs. 2/3 endopod length from apex; endopods of male pleopods 2–5 each with 6 vs. 9 segments.

*Mysidopsissuedafrikana* from littoral sandy bottoms on the Atlantic coast of South Africa (Wooldridge 1988), 33–34°S, the only immature specimen known differs from the new species by a telson with bare lateral margins (vs. with 3–5 spines each), terminal margin transverse with three vs. one pair of spines, namely two pairs of large paramedian spines plus a pair of small spines on the disto-lateral edge; antennal scale length 2.25 vs. 2.6–3.7 times maximum width; endopod of uropod with two vs. 3–4 spines below statocyst near mesial margin. Adult males unknown.

*Mysidopsisiluroensis* from sandy *Posidonia* stands in 17–21 m depth off Mataró coast in the Balearic Sea, Mediterranean, 42°N (San Vicente 2013), differs from the new species by a linguiform, terminally truncate telson with broadly rounded disto-lateral edges vs. trapeziform telson with angular edges; each lateral margin all along with 8–10 spines vs. total of 3–5 spines on basal ≤ 2/3 telson length; endopod of uropod with 6–8 spines ventrally between statocyst and 1/3 endopod length from apex vs. 3–4 spines below statocyst near mesial margin; exopod of male pleopod 4 without modified seta vs. presence of large modified seta.

### Color diversity

The spectrum of colour variants of *M.jenseni* Price, 2024, from the E-Pacific shows most striking coincidences with that of the new species from the Canaries. Not considering the lappets on the posterior margin of the carapace and a few additional features in *M.jenseni*, one could almost confound the in-situ images in figs. 1B−D of Price (2024) with those of the new species given in same order (Fig. 1A, C and Fig. 3A). Zimmer (1909) described *M.gibbosa* as light to dark brown, occasionally almost black. Wittmann and Stagl (1996) observed strikingly dark specimens resting on leaves of the seagrass *Cymodocea* or swimming between the leaves. This points to a strong similarity with the black variant of the new species given in Fig. 2C, in fact with leaves of *Cymodocea* in the foreground.

Two South African species, *M.zsilaveczi* Wittmann & Griffiths, 2014 and *M.abbreviata* Wittmann & Griffiths, 2018, show many colour variants, each highly different from that of *M.jenseni* and the new species. This shows that the observed colour patterns are not a general characteristic of the genus. Rather, there is strikingly detailed convergence between an E-Pacific and an E-Atlantic species. *Mysidopsisabbreviata* shows a strong capability for colour change, imitating even the colour patterns of the substrate. This also holds true for the specimen of the new species in Fig. 2A–B, while decisively not so (at least for the human eye) for the specimens in Fig. 1A and Fig. 2C. Based on current knowledge, several species of the genus *Mysidopsis* show the greatest capability of colour change in the Mysidae (Wittmann and Griffiths 2018) observed from tidal pools to the shallow sublittoral, mostly under conditions of bright light. So far, the physiological and genetic factors limiting the spectrum of colour change are unknown.

### Biogeography

The distribution of the new species at numerous stations, so far known exclusively in the Canary and Cape Verde archipelagos, supports the classical biogeographical concept of Macaronesia which comprises the Azores, Madeira, Selvagens, Canary and Cape Verde archipelagos. In line with this, excursions to the coast of Senegal (West Africa) and to the Gulf of Guinea did not yield this species which, however, was also not found upon several excursions to Madeira. Of course, a single species does not constitute a biogeographical unit; nonetheless, its distribution should be integrated in future statistics and biogeographical considerations. By contrast to the present findings on this particular species, recent trends point to a division of Macaronesia: upon a review based on 230 literature references, Spalding et al. (2007) concluded that the biota of the Cape Verdes differs significantly from the other Macaronesian archipelagos and appears to be a sub-province within the West African Transition province. According to Freitas et al. (2019), the remaining archipelagos may belong to the Lusitanian province.

## Supplementary Material

XML Treatment for Mysidopsis canariensis
